# Predicting Participation Willingness in Ecological Momentary Assessment of General Population Health and Behavior: Machine Learning Study

**DOI:** 10.2196/41412

**Published:** 2023-08-02

**Authors:** Aja Murray, Anastasia Ushakova, Xinxin Zhu, Yi Yang, Zhuoni Xiao, Ruth Brown, Lydia Speyer, Denis Ribeaud, Manuel Eisner

**Affiliations:** 1 Department of Psychology University of Edinburgh Edinburgh United Kingdom; 2 Centre for Health Informatics Lancaster University Lancaster United Kingdom; 3 Clinical Psychology Department University of Edinburgh Edinburgh United Kingdom; 4 Department of Psychology University of Cambridge Cambridge United Kingdom; 5 Jacobs Center for Productive Youth Development University of Zurich Zurich Switzerland; 6 Institute of Criminology University of Cambridge Cambridge United Kingdom

**Keywords:** ecological momentary assessment, experience sampling, machine learning, recruitment, sampling

## Abstract

**Background:**

Ecological momentary assessment (EMA) is widely used in health research to capture individuals’ experiences in the flow of daily life. The majority of EMA studies, however, rely on nonprobability sampling approaches, leaving open the possibility of nonrandom participation concerning the individual characteristics of interest in EMA research. Knowledge of the factors that predict participation in EMA research is required to evaluate this possibility and can also inform optimal recruitment strategies.

**Objective:**

This study aimed to examine the extent to which being willing to participate in EMA research is related to respondent characteristics and to identify the most critical predictors of participation.

**Methods:**

We leveraged the availability of comprehensive data on a general young adult population pool of potential EMA participants and used and compared logistic regression, classification and regression trees, and random forest approaches to evaluate respondents’ characteristic predictors of willingness to participate in the Decades-to-Minutes EMA study.

**Results:**

In unadjusted logistic regression models, gender, migration background, anxiety, attention deficit hyperactivity disorder symptoms, stress, and prosociality were significant predictors of participation willingness; in logistic regression models, mutually adjusting for all predictors, migration background, tobacco use, and social exclusion were significant predictors. Tree-based approaches also identified migration status, tobacco use, and prosociality as prominent predictors. However, overall, willingness to participate in the Decades-to-Minutes EMA study was only weakly predictable from respondent characteristics. Cross-validation areas under the curve for the best models were only in the range of 0.56 to 0.57.

**Conclusions:**

Results suggest that migration background is the single most promising target for improving EMA participation and sample representativeness; however, more research is needed to improve prediction of participation in EMA studies in health.

## Introduction

### Overview

Ecological momentary assessment (EMA) is a technique for capturing the experiences of participants—including momentary cognitions, emotions, behaviors, and events—in the flow of their daily lives [1]. Factors such as increases in smartphone ownership over the past years have made EMA highly feasible and have led to an explosion in its popularity for studying mental and physical health [2]. It is argued to be particularly valuable in adolescents and young adults owing to the fact that smartphone use tends to be high and well-embedded in the daily routines of young people. Although the technique has led to clear advances in our understanding of the daily life dynamics of a range of not only health but also social, psychological, and behavioral phenomena, the majority of EMA research remains based on convenience samples [3]. This leaves open the possibility that EMA samples are selective with respect to characteristics that are under study (eg, mental health and substance use) and could mean that the parameter estimates relating to these variables are biased with respect to the underlying target population. In contrast, knowledge of the factors that predict participation in EMA studies may help inform recruitment and measurement strategies that mitigate these biases and maximize recruitment success overall. The goal of this study was, therefore, to leverage a well-characterized participant pool ascertained to be a young adult community to explore the extent to which willingness to participate in EMA studies in the young adult general population is dependent on respondent characteristics (ie, nonrandom). We also sought to identify which specific respondent characteristics were the potentially most important predictors.

Though there has been considerable attention paid to optimizing the EMA methodology, including the way that data are collected, analyzed, and reported [4], a major potential weakness of the existing EMA literature is its reliance on a convenience sampling approach. A comprehensive systematic review of the EMA literature [3] found that out of the 496 studies included in their analysis 296 (60%) used a convenience sampling approach, whereas only 53 (11%) used representative samples and 109 (22%) used college student samples. Although convenience sampling may be adequate in some contexts (eg, where the focus is methods development, feasibility, or piloting), it has important drawbacks compared to methods such as simple random sampling or probability sampling. Foremost among these is that it carries the risk that the sample drawn is not representative of the underlying target population and thus parameters estimated in that sample are biased with respect to the values in that population. Furthermore, if no information is available on the sample characteristics relative to the underlying target population, there is no possibility to correct this lack of representativeness, for example, using weighted analyses [5]. Given this, it is important to illuminate the extent to which EMA samples may be selective with respect to respondent characteristics. Respondent characteristics that are often the subjects of study in EMA designs, such as mental health symptoms, substance use, physical health, and health behaviors [2,6-8], are particularly important to explore because bias will be most problematic when sample selectivity is related to the same characteristics about which a study seeks to make inferences [9].

A further advantage of illuminating the factors that predict participation in EMA studies is that this knowledge may be able to inform recruitment and measurement strategies to maximize participation and minimize recruitment bias [10,11]. For example, if individuals high in certain characteristics from the population (eg, antisocial behavior, mental health symptoms) have a lower likelihood of participating in EMA studies, then such individuals could be oversampled and more time and resources could be budgeted for their recruitment, given that they may require more efforts to recruit or larger incentives to motivate participation [11]. The characteristics that are related to participation may also provide insights into the mechanisms that determine participation, and this could inform how the study is presented to participants. For example, tailored presentations of the study highlighting different aspects could be developed depending on participant profiles [12]. Predictors of participation can also inform customized protocols for participants of different profiles. For example, participants predicted to have a lower likelihood of participating could be offered a lower burden variant of the protocol to help ensure that some information is gathered on participants who might otherwise decline to participate altogether [11]. However, where a representative sample is not viewed as important, participants with a high probability of participation could be targeted to minimize the resources consumed by pursuing the recruitment of participants who are unlikely to take part.

Much has been learned about predictors of participation in health and broader research studies in general, suggesting that a wide range of sociodemographic, individual, and social and situational characteristics may be related to participation and attrition [13-15]; however, despite the potential value of information on predictors of participation in EMA studies, there has been very little research on this topic specifically. It is important to illuminate predictors of participation in EMA studies as a specific type of research design as it possesses some unique characteristics, including its reliance on the use of smartphone-based technologies, the prolonged and intensive data collection schedule, and the collection of data in the flow of people’s daily lives. These characteristics might create additional barriers to participation, perhaps especially in more socially disadvantaged groups [16]. One study examined the influence of study design characteristics on respondents’ ratings of their likelihood of agreeing to participate in a hypothetical EMA study [17] and found that participants were more likely to report willingness to take part in studies that were shorter in duration, with fewer prompts, and with more generous incentives. However, no study to our knowledge has examined the predictors, including respondent characteristics, that are predictive of taking part in real EMA studies.

Some indications of the factors that may be important in predicting EMA study participation come from studies that have explored a range of respondent characteristics of EMA compliance conditional on agreeing to participate [18-22]. These characteristics have included gender, age, mental health, substance use, and antisocial traits; however, results have not always been replicated across studies and are based on only a single or handful of samples. As such, there remains considerable uncertainty around which—if any—respondent characteristics are consistent and important predictors of compliance. Furthermore, it cannot be assumed that the same factors will drive initial participation willingness and compliance once enrolled.

### This Study

Given the value of illuminating the respondent characteristics associated with participation in EMA studies, we here explored a wide range of candidate predictors of willingness to participate in the Decades-to-Minutes (D2M) EMA study. We leveraged the fact that the D2M study is embedded within the longitudinal Zurich Project on the Social Development from Childhood to Adulthood (z-proso) study [23], providing 2 major advantages. First, the z-proso sample was drawn as a stratified random sample from a school year cohort of Switzerland’s largest city and has suffered little nonrandom attrition over time [14]. Second, the z-proso study provides comprehensive respondent characteristic information on both participants who consented to be contacted about the EMA study and those who did not. This means that we have the rare advantage of having rich information on both EMA respondents and nonrespondents. We used 2 complementary approaches to evaluate predictors of consenting to be contacted about the EMA study: logistic regression and tree-based machine learning methods. Given the lack of a standardized approach for examining the predictors of research participation, previous studies have recommended these approaches be used in conjunction, as they can provide more comprehensive insights into relevant factors [24]. Findings from both are reported because logistic regression can provide greater interpretability, but the tree-based methods are likely to give higher accuracy and also provide complementary information on important predictors through variable importance metrics.

The respondent characteristics explored as potential predictors of consenting to be contacted about an EMA study were informed by previous studies suggesting possible links between factors, such as sociodemographic characteristics, mental and physical health, well-being, prosocial and antisocial traits, and compliance with EMA protocols [15,20]. However, because machine learning approaches allow the exploration of a large number of features, we also included several more exploratory predictors that could in principle be related to consenting to participate in an EMA study or that may be of interest in EMA studies of health outcomes and behaviors (and where selective sampling is thus of greatest concern for health research). Our goal was to examine the extent to which participation can be predicted based on respondent characteristics and to identify the most important specific predictors.

## Methods

### Participants

Participants were drawn from the age-20 data collection wave of z-proso [23]. Z-proso is a longitudinal cohort study of child psychosocial development with mental health as a prominent topic [25-28]. The study began in 2004 and recruited children entering primary school in Zurich, Switzerland, at a median age of 7 years. Sampling was conducted via a stratified random sampling procedure that considered school size and location (with the goal of ensuring adequate representation of children from diverse sociodemographic backgrounds). Since then, the participants have been followed up at ages 8, 9, 10, 11, 12, 13, 15, 17, and 20 years. A further set of data collection waves was also completed to gather information specifically on the impact of the COVID-19 pandemic [29]. Overall, 1572 of the original target sample of 1675 had provided data for at least one measurement wave, and previous analyses of response and attrition have suggested that the sample achieves a good representation of the target sample [14]. The z-proso study also has several substudies (see Silvia et al [22] for an overview); our study concerns willingness to participate in the D2M EMA substudy [30,31].

The participants in the current sample were those who were recruited at the age-20 wave of z-proso (n=1183). Of this sample, 979 had complete data on the below-described predictors and outcomes. See Table S1 and Table S2 in Multimedia Appendix 1 [32-44] for the descriptive statistics for these samples.

### Procedure

In the main wave of data collection for the z-proso study, taking place when participants were aged 20 years, participants were informed of add-on studies in which they could take part. Among these studies was the D2M study [31]: a 2-week EMA study. Participants were provided with a brief description of the study, including information on the study lead, the purpose of the study, what would be involved, the incentive being offered, and contact information for the researchers if they had any questions. The study was framed as a study of everyday experiences that make people feel bad or good or that might cause them to show aggression. They were also informed that if they agreed to potentially participate, they would receive further information and instructions later by email. A record was kept for all z-proso participants in the age-20 wave regarding whether they consented to be contacted again later to take part in D2M. A separate, more comprehensive information sheet and an informed consent form were provided to participants at the point at which the D2M study took place.

Further information on the EMA study itself can be found in previous publications [30,31]. In brief, it involved 2 weeks of smartphone-based data collection in which participants were prompted to complete short surveys 4 times a day at quasi-random intervals over a 14-day period. The measures asked participants about their momentary stress, emotions, aggressive behavior, provocations, substance use, and context (activity and who they were with) and were designed to take no more than 2 minutes each time.

### Measures

Full details of the measures are included in Multimedia Appendix 1 [32-44]. Each predictor was chosen based on data availability within z-proso, and the selections for the current analyses were comprehensive based on the exploratory goal of the study, that is, all available measures within each domain were used provided the data quality was deemed to be sufficiently good (ie, reliable measures and no excessive missingness). In cases where measures were highly redundant, only one was selected. The rationale for the selection of the measures for the z-proso study itself is discussed by Silvia et al [22].

Sociodemographic predictors were gender and socioeconomic status, based on the International Socioeconomic Index of occupational status (ISEI) measures of household occupational prestige [32], educational level, and migration background (1=at least 1 parent born in Switzerland, 2=both parents born abroad) self-reported by youth aged 11 to 12 years.

Mental health predictors were symptoms at age 20 years in the domains of attention deficit hyperactivity disorder (ADHD) symptoms and depression, anxiety, and psychosis-like symptoms measured with the Social Behavior Questionnaire (SBQ; [33,34]). Psychosis-like symptoms were measured using an abbreviated 6-item version of the Community Assessment of Psychic Experiences Scale [35]. Suicidal ideation (“I thought about killing myself”) and self-injury (both including suicidal and nonsuicidal self-injury: “I harmed myself on purpose [e.g., cut my arm, tore wounds open, hit my head, tore out my hair”]) were both measured with single items.

Psychological well-being predictors were hope, self-control, self-efficacy, optimism, general trust, perceived stress, and future orientation. Hope was measured with an abbreviated version of the Adult Hope Scale [36]. Self-control was measured by a 10-item adapted version of the Grasmick Low Self-Control Scale [45]. Self-efficacy was measured with 5-item adaptation of the scale proposed by Schwarzer and Jerusalem [37]. Optimism was measured with 4 items (ie, “I’m happy,” “life is beautiful,” “I’m full of energy,” and “I laugh often”). Perceived stress was measured using a 4-item version of the Perceived Stress Scale [38]. Future orientation was measured with 5 items, covering the vividness of the future (eg, “easy to imagine future”), connectedness with the future (“level of connection between past or present with future”), and feelings about the future (“feel about future self”).

Social well-being predictors were social exclusion, social support from a trusted adult, and bullying victimization. Social support was measured with 5 items (eg, “adults to talk about problems,” “adults I admire,” “discuss my problems with adults,” “adults I can trust,” “adult social support”). Social exclusion was measured with 6 items (eg, “not feeling as part of society,” “being segregated,” “no chance in this society,” “feeling that others depreciate me,” “feeling alienated,” “feeling worthless for society”). Bullying victimization was measured using the 4-item Zurich Brief Bullying Scale, which captures bullying victimization in various forms, including physical and verbal aggression [39].

Physical predictors were general health and BMI. General health was measured by a single item asking youth to rate their general health. BMI was derived from self-reported height and weight at the age of 20 years.

Prosocial trait predictors were general trust, prosociality, and moral shame. Prosociality was measured by 10 SBQ items, capturing both prosocial emotions (eg, empathy) and helpful behaviors. General trust was measured using a 3-item (eg, “most people can be trusted”) measure adapted from the World Values Survey [40]. Moral shame was measured by 3 items (eg, “ashamed after lying,” “feel bad when doing wrong,” and “guilty when done wrong”).

Antisocial trait predictors at the age of 20 years were delinquency, bullying perpetration, aggression, substance use, psychopathy, and violent ideations. Delinquency was measured by 24 items. Bullying perpetration was measured using the 4-item Zurich Brief Bullying Scale perpetration scale [39]. Items were analogous to those in the victimization scale but presented from a perpetrator’s perspective. Substance use was measured by 4 items capturing substance use over the previous 12 months on a 6-point scale from never to daily (never, once, 2 to 5 times, 6 to 12 times monthly, 13 to 52 times weekly, and 53 to 365 times daily). One item captured the use of tobacco, 1 captured alcohol use (beer and alcopops), 1 captured alcohol use (spirits) and 1 captured cannabis use. Violent ideations were measured using the expanded Violent Ideations Scale [41,42], capturing fantasies of committing acts of physical, indirect, and sexual violence across 17 items. Aggression was measured using 19 items from the SBQ, covering physical, indirect, proactive, and reactive aggression.

### Statistical Procedure

We used 2 main complementary approaches to evaluate predictors of consenting to participate in the D2M EMA study, all of which were implemented in R [46]: logistic regression and tree-based machine learning techniques, specifically, classification and regression tree (CART) and random forest.

In this study, we fit a series of univariate logistic regression models to describe the unadjusted effects of each predictor on the outcome. We then fit a multiple logistic regression model to estimate the effects of each predictor, mutually adjusting for all others. For BMI, both linear and quadratic effects were included, but for all other predictors, only linear effects were included. These were implemented using the *glm( )* function.

Second, we used tree-based machine learning approaches. We began with a CART model. CART is based on identifying partitions of the data on the basis of predictors to maximize the homogeneity of the outcome within the partitions. A major benefit of CART is that it can allow for complex interactions between large numbers of predictors. Unlike logistic regression, CART does not rely on strict assumptions about the form of the relations between predictors and outcomes. We allowed the algorithm to proceed up to a stopping criterion point set to a minimum group size of 20. As CART produces only a single tree, we were able to visualize this tree to aid in our interpretation of the findings. This was the primary purpose of fitting CART in this study, given that random forest analysis is a closely related but typically superior method for prediction. The CART model was implemented using the *rpart ( )* function from the *rpart* package [47].

As noted, though the fitting of a single tree in CART has the advantage of easy interpretability, random forest analysis has been shown to generally improve the predictive power of CART. Thus, we also used a random forest approach, fitting 500 classification trees. Random forest [48] is an ensemble method that can improve on CART by addressing some of its limitations. In CART, a single tree is produced, making it easy to interpret but suboptimal in its performance. In random forest analysis, many trees are fit and combined to optimize prediction [49,50]. Random forest proceeds by drawing a large number of bootstrap samples (with replacement) and applying a CART-like algorithm to fit a tree in each. However, the algorithm considers only a random sample of features (predictors) at each partition to help to decorrelate the trees. Following the fitting of a large number of trees, the entire ensemble can be used to predict the outcome of interest based on combining the predictions of the trees. The number of features considered in each tree can impact findings. Although a commonly used heuristic suggests allowing m = √p predictors for each tree, where p is the total number of predictors, it is recommended that several variations are attempted. We, therefore, allowed the number of features randomly selected per tree to act as a tuning parameter, with m=2, 5, and 10 trialed. Each bootstrap sample was drawn with replacement and to a size equaling the size of the initial data being sampled. This was implemented using the *rf(* ) function for random forest analysis from the *caret* package [51]. For the best-performing random forest model (see Comparison of Approaches section) based on area under the curve (AUC), we also calculated feature importance for our variables, using a permutation approach [52] in which the decrease in model performance when each feature is randomly permuted is measured. We measured the decrease in performance using the root mean squared error (RMSE) and this difference was used as a metric of feature importance. Educational level was omitted from this analysis because the permutation method only supports features that can be represented as numeric.

### Comparison of Approaches

We calculated predictive performance metrics for each model to evaluate model adequacy and compare performance across models. For this, we used 5- and 10-fold cross-validation and calculated accuracy, AUC, κ, the *F_1_*-statistic, sensitivity, specificity, recall, precision, positive predictive value, negative predictive value, and cross-validation AUC.

AUC was used as the main measure of model adequacy. It measures classification accuracy based on the area under a receiver operator characteristic curve and takes values between 0.50 and 1, with 0.50 to 0.60 conventionally representing no discrimination and 0.90 to 1 representing “outstanding.” We also considered the κ values as a global measure of model adequacy that provides a measure of accuracy (from 0=no agreement to 1=perfect agreement) taking into account correct prediction by chance alone. Conventional interpretations of κ run from <0.20=poor agreement up to 0.80-1=very good agreement. The other metrics are provided for additional information about model performance.

Both 5- and 10-fold cross-validation were used because, although the latter is often recommended, given the small sample size of this study, it was judged that it may be beneficial to additionally trial a cross-validation split that used larger training samples. All statistics are presented for information, but AUC and cross-validation AUC were used as the primary basis for comparisons across approaches. For these analyses, the *mikropml* [53] package in R was used to automate the model training and evaluation pipeline. Here, we also included a tuning step for the logistic regression and CART analyses drawing on *glmnet( )* from the *glmnet* package [54] for penalized logistic regression (the penalization helps provide more sparse or parsimonious solutions in multipredictor models) and *rpart2( )* from the *rpart* package for CART. For the penalized logistic regression, a complexity penalty λ was used as a tuning parameter (α was kept constant at 0 and λ varied between 0 and 10), and for CART the maximum depth of the tree (range 1-30) was used for tuning. The AUC was used to select the best model from those tried out. To guard against overfitting, a training to validation split of 80% to 20% was used.

### Missing Data Strategy

Given that our data set included some missing data, we also repeated the procedure using 2 alternative methods of dealing with missingness: listwise deletion (complete case analysis) and single imputation. The latter was conducted using a multivariate chained equations approach using the *mice* package in R [55]. Single imputation was used rather than multiple imputation given the practical difficulties of combining multiple imputation with techniques, such as CART and random forest. Specifically, a fully conditional specification approach was used with categorical and continuous variables imputed using predictive mean matching for continuous, polytomous regression for categorical predictors with more than 2 categories, and logistic regression for binary predictors and the participation outcome.

### Ethics Approval

Ethical approval for these studies was obtained from the University of Zurich’s Faculty of Arts and Social Science’s Ethics Committee (Nr. 2018.2.12). Written informed consent was obtained from participants prior to data collection.

## Results

### Descriptive Statistics

There were 599 participants who were not willing in principle to participate in D2M and 584 (49.4%) who were. Descriptive statistics for the predictors are provided in Table S1 in Multimedia Appendix 1 [32-44]. For the subsample of participants with complete cases, 493 (50.3%) were not willing in principle to participate in D2M, while 486 (49.6%) were. Descriptive statistics for this subsample are provided in Table S2 in Multimedia Appendix 1 [32-44].

### Logistic Regressions

The unadjusted effects for each predictor based on separate logistic regressions for each predictor are provided in Table 1. In these models, gender, migration status, anxiety symptoms, ADHD symptoms, and prosociality were the only significant predictors. Specifically, the likelihood of agreeing to be contacted about a future EMA study was higher for female respondents, for respondents with at least 1 parent born in Switzerland, and for respondents with higher levels of anxiety symptoms, ADHD symptoms, stress, and prosociality.

The adjusted effects for each predictor based on a multiple logistic regression model including all predictors simultaneously are provided in Table 2. Because the complete case and single imputation analyses yielded similar findings, only the latter are presented, with results for the former presented in Table S3 in Multimedia Appendix 1 [32-44]. In this model, migration status, social exclusion, and tobacco use were significant predictors of willingness to participate in an upcoming EMA study. Specifically, participants for whom at least 1 parent was not born in Switzerland, those who had higher levels of tobacco use, and those who reported higher levels of social exclusion were less likely to agree to be contacted. The main difference between the complete case and single imputation analysis was that prosociality was not significant in the latter but was in the former.

**Table 1:** Unadjusted effects for each predictor from logistic regression. Reference category for gender is “male,” reference category for migration status is “at least 1 parent born in Switzerland,” reference category for education is “incomplete compulsory school.” Italics indicate statistical significance at *P*<.05; B indicates the regression coefficient.

Predictors				B		SE		*P* value		Odds ratio (95% CI)
Gender				0.343		0.117		.*03*		1.409 (1.120-1.772)
Migration background				−0.428		0.118		*<.001*		0.652 (0.517-0.821)
International Socioeconomic Index of occupational status				0.002		0.003		.43		1.002 (0.996-1.008)
**Education**										
	Compulsory school, elementary vocational training		0.469		0.291		.11		1.599 (0.904-2.827)	
	Domestic science course, 1-year school of commerce		0.379		0.561		.50		1.462 (0.486-4.387)	
	Apprenticeship		0.250		0.285		.38		1.284 (0.734-2.245)	
	Full time vocational school		0.146		0.399		.72		1.157 (0.529-2.530)	
	A-levels		0.392		0.299		.19		1.480 (0.824-2.659)	
	Vocational high education		0.246		0.392		.53		1.279 (0.593-2.757)	
	Technical school or vocational college		0.168		0.414		.68		1.183 (0.525-2.663)	
	Vocational high school, higher specialized school		0.542		0.417		.19		1.719 (0.759-3.894)	
	University		0.468		0.282		>.99		1.597 (0.919-2.775)	
Depression				0.012		0.008		.14		1.012 (0.996-1.028)
Anxiety				0.043		0.016		*.01*		1.044 (1.012-1.077)
Psychosis-like symptoms				0.018		0.019		.34		1.018 (0.981-1.057)
Attention deficit hyperactivity disorder symptoms				0.025		0.009		*.01*		1.025 (1.007-1.044)
Hope				−0.112		0.129		.38		0.894 (0.694-1.151)
Self-efficacy				−0.155		0.125		.21		0.857 (0.670-1.094)
Optimism				0.009		0.025		.74		1.009 (0.961-1.060)
Stress				0.033		0.016		*.04*		1.033 (1.002-1.066)
Future orientation				0.009		0.014		.52		1.009 (0.982-1.037)
Social support				0.021		0.022		.35		1.021 (0.978-1.066)
Social exclusion				−0.009		0.016		.57		0.991 (0.960-1.023)
Bullying victimization				0.154		0.124		.22		1.166 (0.915-1.487)
General health				0.000		0.003		.94		1.000 (0.994-1.006)
BMI				−0.085		0.080		.29		0.918 (0.785-1.074)
BMI^2^				0.001		0.001		.53		1.001 (0.999-1.003)
General trust				0.171		0.088		.05		1.186 (0.999-1.410)
Shame				0.169		0.090		.06		1.184 (0.993-1.413)
Prosociality				0.028		0.011		*.01*		1.028 (1.006-1.051)
Bullying perpetration				0.148		0.134		.27		1.160 (0.892-1.508)
Tobacco use				−0.057		0.030		.06		0.945 (0.891-1.002)
Cannabis use				0.039		0.035		.26		1.040 (0.971-1.114)
**Alcohol use**										
		Beer, wine, alcopops	0.071		0.042		.09		1.073 (0.989-1.166)	
		Spirits	0.043		0.047		.36		1.044 (0.952-1.145)	
Aggression				−0.003		0.008		.69		0.997 (0.981-1.013)
Delinquency				0.039		0.027		.14		1.040 (0.986-1.096)
Violent ideations				0.002		0.012		.89		1.002 (0.979-1.026)

**Table 2:** Multiple logistic regression model results based on single imputation. The reference category for gender is “male,” the reference category for migration status is “at least 1 parent born in Switzerland,” and the reference category for education is “incomplete compulsory school.” Italics indicate statistical significance at *P*<.05.

Categories		Estimate	SE	*P* value	Odds ratio (95% CI)
Intercept		0.278	0.373	.46	1.321 (0.636-2.743)
Gender		0.061	0.037	.09	1.063 (0.989-1.143)
Migration background		−0.106	0.035	*.003*	0.899 (0.840-0.963)
**Education**					
	Compulsory school/elementary vocational training	0.101	0.069	.14	1.107 (0.966-1.266)
	Domestic science course/1-year school of commerce	0.033	0.137	.81	1.033 (0.790-1.352)
	Apprenticeship	0.014	0.070	.85	1.014 (0.884-1.163)
	Full time vocational school	−0.001	0.095	>.99	0.999 (0.829-1.203)
	A-levels	0.051	0.075	.50	1.052 (0.908-1.219)
	Vocational high education	0.036	0.098	.71	1.037 (0.855-1.256)
	Technical school or vocational college	0.026	0.100	.80	1.026 (0.844-1.249)
	Vocational high school/higher specialized school	0.088	0.106	.41	1.092 (0.887-1.344)
	University	0.087	0.079	.27	1.091 (0.934-1.274)
International Socioeconomic Index of occupational status ISEI		−0.002	0.001	.08	0.998 (0.996-1.000)
Depression		-0.001	0.004	.86	0.999 (0.991-1.007)
Anxiety		0.004	0.007	.55	1.004 (0.990-1.018)
Psychosis-like symptoms		0.007	0.006	.29	1.007 (0.995-1.019)
Attention deficit hyperactivity disorder symptoms		0.004	0.003	.27	1.004 (0.998-1.010)
Hope		−0.038	0.050	.46	0.963 (0.873-1.062)
Self-efficacy		−0.045	0.046	.32	0.956 (0.874-1.046)
Optimism		0.004	0.009	.68	1.004 (0.986-1.022)
Stress		0.004	0.007	.60	1.004 (0.990-1.018)
Future orientation		0.005	0.004	.21	1.005 (0.997-1.013)
Social support		0.003	0.006	.69	1.003 (0.991-1.015)
Social exclusion		−0.013	0.006	*.04*	0.987 (0.976-0.999)
Bullying victimization		0.014	0.043	.75	1.014 (0.932-1.103)
Health		0.001	0.001	.46	1.001 (0.999-1.003)
BMI		−0.003	0.020	.88	0.997 (0.959-1.037)
BMI^2^		0.000	0.000	.94	1.000 (1.000-1.000)
Trust		0.017	0.025	.49	1.017 (0.969-1.068)
Shame		0.003	0.026	.92	1.003 (0.953-1.055)
Prosociality		0.005	0.003	.08	1.005 (0.999-1.011)
Bullying perpetration		0.029	0.046	.53	1.029 (0.941-1.127)
Aggression		−0.002	0.003	.43	0.998 (0.992-1.004)
Delinquency		0.008	0.008	.32	1.008 (0.992-1.024)
Violent ideations		0.002	0.004	.61	1.002 (0.994-1.010)
Tobacco		−0.019	0.009	*.04*	0.981 (0.964-0.999)
Beer		0.012	0.018	.51	1.012 (0.977-1.048)
Spirits		−0.005	0.019	.78	0.995 (0.959-1.033)
Cannabis		0.016	0.011	.15	1.016 (0.994-1.038)

### CART model

The CART model was fit to the single imputation data [56]. In this model, the first partition was based on migration background with the next partitions based on tobacco use and prosociality. Partitions based on psychosis-like symptoms, BMI, health, and self-efficacy occurred at the next level. The CART model was also fit to the complete case data [57] and yielded similar partitions at the highest levels of branching.

### Random Forest

In the random forest analysis with complete cases, the model with 2 predictors randomly selected for each tree provided the best AUC of the different random forest analyses trialed, and there was little difference between the model fit to the complete case versus single imputation data set. Model evaluation metrics for this model are provided in Table 3, with further metrics provided in Table S4 of Multimedia Appendix 1 [32-44]. These suggested that willingness to participate in an upcoming EMA study was only weakly predicted by the random forest models fit based on 30 respondent characteristics predictors. For example, the AUC values for the complete case and single imputation data sets were only 0.585 and 0.576, respectively (on a scale of 0.50 to 1, with 0.5 indicating chance-level prediction and 1 indicating perfect prediction). The cross-validation AUCs estimating performance in new data were similarly low at 0.569 and 0.562 for the complete case and single imputation analyses, respectively.

**Table 3:** Model evaluation metrics for performance comparison with tuning and cross-validation.

Model	Data	CV folds	Tuning parameters^a^	AUC^b^	κ	PPV^c^	NPV^d^	Precision	Recall	CV^e^ AUC
Logistic	CC^f^	10	λ=1	0.594	0.159	0.574	0.586	0.574	0.632	0.557
CART^g^	CC	10	Tree depth=16	0.601	0.159	0.591	0.570	0.591	0.531	0.542
RF^h^	CC	10	Features/split=2	0.585	0.035	0.518	0.518	0.518	0.592	0.569
Logistic	SI^i^	10	λ=1	0.572	0.071	0.537	0.535	0.537	0.613	0.556
CART	SI	10	Tree depth=16	0.557	0.115	0.564	0.551	0.564	0.555	0.533
RF	SI	10	Features/split=2	0.576	0.095	0.545	0.556	0.545	0.664	0.562
Logistic	CC	5	λ=1	0.594	0.159	0.574	0.586	0.574	0.633	0.556
CART	CC	5	Tree depth=30	0.601	0.159	0.591	0.570	0.591	0.531	0.535
RF	CC	5	Features/spilt=2	0.613	0.179	0.585	0.596	0.585	0.633	0.568
Logistic	SI	5	λ=10	0.569	0.020	0.512	0.545	0.512	0.916	0.551
CART	SI	5	Tree depth=16	0.566	0.072	0.540	0.531	0.540	0.563	0.522
RF	SI	5	Features/split=2	0.570	0.121	0.556	0.570	0.556	0.664	0.557

^a^Parameters selected based on the highest AUC across models were compared.

^b^AUC: area under the curve.

^c^PPV: positive predictive value.

^d^NPV: negative predictive value.

^e^CV: cross-validation.

^f^CC: complete case.

^g^CART: classification and regression tree.

^h^RF: random forest.

^i^SI: single imputation.

Results of the permutation importance analysis from the random forest with 2 features per split suggested that migration had the largest importance (0.004 reduction in RMSE), followed by BMI, hope, and prosociality (all 0.002 reduction).

### Comparison of Approaches

For comparison with the random forest analysis, the model evaluation (including model adequacy) metrics for multiple logistic regression and CART, including a tuning step and cross-validation, are also provided in Table 3. These metrics suggested that random forest analysis was only slightly better in terms of cross-validation AUC and performed slightly worse than other models on some metrics. For example, CART with a maximum tree depth of 16 had a slightly higher AUC (0.601) than a random forest considering 2 features per split when fit into the complete case (0.585) and using 10-fold cross-validation. Furthermore, logistic regression showed a higher cross-validation AUC than CART in both the complete case (0.557 vs 0.542) and single imputation (0.556 vs 0.535) data sets using 10-fold cross-validation and 5-fold cross-validation (0.556 vs 0.535 for complete case and 0.551 vs 0.522 for single imputation).

## Discussion

### Overview

The purpose of this study was to examine the extent to which being willing to participate in EMA research is related to respondent characteristics and to identify the most critical predictors of participation. Considering the findings from both logistic regression and tree-based machine learning approaches, migration background, social exclusion, tobacco use, and prosociality emerged as the most important predictors, with gender, anxiety, and stress also being significantly related to participation willingness in univariate logistic regression analyses. These characteristics represent potential targets in terms of designing recruitment, measurement, and analysis strategies to mitigate sampling-based biases in EMA studies. Overall, however, participation was only very weakly predictable from respondent characteristics (AUCs between 0.56 and 0.60), with random forest analysis and CART providing only a minimal increase in performance relative to logistic regression.

Our findings are consistent with previous research suggesting that migration status is associated with lower research study participation. In the broader z-proso sample, for example, an earlier study found that youth whose parents’ first language was one of several languages other than German (indicative of a migrant background) were less likely to participate at baseline. A similar pattern has been observed in other (non-EMA) European studies and is consistent with the broader underrepresentation and lower response rates of socially disadvantaged groups in social science and medical research [15,58,59]. We similarly identified a significant effect of social exclusion on participation willingness in this study, which held after adjusting for migration status (and all other predictors). While it has been noted that part of the reason for the underrepresentation of socially disadvantaged groups in research may relate to insufficient efforts to recruit participants from diverse backgrounds (eg, Mullarkey et al [60]), our findings also highlight greater reluctance on the participants’ side. Possible reasons may include a lack of trust in the research process, potentially relating to historical mistreatment or based on a feeling that the research may not benefit their community [61]. This concern may be exacerbated for data collection methods such as EMA, which, by collecting data through participants’ smartphones and embedding this data collection within their daily routines, may feel more intrusive.

Some previous studies have explored potential strategies for encouraging the participation and retention of socially disadvantaged groups in research [60,61]. Alongside generic methods for recruiting difficult-to-recruit respondents [11], recommended strategies have included community engagement, co-design approaches, developing and using a systematic contact plan, adaptation and translation of study materials, the use of interviewers from the community, and the training of field workers to help them build rapport with potential participants [60-62]. However, further systematic analyses to illuminate the most effective components of interventions to recruit and retain socially disadvantaged groups in research are needed [60]. Overall, it is recommended that greater time and resources be allocated to the recruitment of individuals with a migrant background. These individuals may be more difficult to recruit, but it is essential to ensure they are adequately represented in research.

Tobacco use and prosociality were also highlighted as potential targets for improving participation in EMA health studies. Our findings here add to a somewhat less clear evidence base. For example, some studies that have examined willingness to take part in other research designs, such as biobank research, have found that prosocial traits positively predict willingness, whereas others have found no association [63-65]. Similarly, while there has been some evidence that smoking is related to compliance in EMA studies, there is a lack of research on whether tobacco use represents an influence on willingness to participate in research [6,20]. Further research is, therefore, required to clarify their association with participation willingness.

The fact that participation willingness could be overall only weakly predicted from sociodemographic, health, and behavior-related respondent characteristics suggests that there are other factors to consider alongside these to improve the prediction of participation propensity. Although previous studies have suggested that participation in research may be predicted by a range of sociodemographic, individual, and situational factors [13,15,66], the available evidence base primarily relates to traditional survey methods, and it is not clear how well this generalizes to EMA studies. Additional predictors to consider for EMA research participation willingness could include factors such as how much and in what ways individuals use and engage with their smartphones, work or life schedules (eg, some occupations may preclude the ability to respond to EMA prompts during the working day), and markers of interest in the specific topic of the EMA study.

Nevertheless, the fact that participation willingness was (albeit weakly) associated with respondent characteristics suggests that reliance on convenience samples is likely to result in samples that are somewhat biased with respect to an underlying target population. To mitigate this bias, it may be helpful to draw EMA participants from well-characterized participant pools to help test and correct for sample selection biases [31,67-69]. As illustrated in this study, the availability of data on both nonrespondents and respondents allows for an examination of selective participation. For example, the D2M EMA study recruited participants from the z-proso study, which had an explicit sampling frame and rich data on participant backgrounds [31]. Similarly, in a study of older adults with and without cognitive impairment [70], the authors were able to compare those who agreed to participate in an EMA substudy to those who did not, finding that those who agreed to be part of their control (but not cognitive impairment group) had higher educational levels and cognitive test scores. Furthermore, to help adjust for any such biases, techniques such as data weighting, multiple imputation, full information maximum likelihood, or Bayesian estimation utilizing information from both respondents and nonrespondents can be used under an assumption of missingness at random [71,72].

### Limitations and Future Directions

The primary limitation of this study is that we used participation willingness as a proxy for participation. This was due to the fact that, owing to resource constraints, it was ultimately not possible to include all willing participants in the EMA study. Furthermore, while the main z-proso study has shown little evidence of nonrandom attrition, it is slightly selective relative to the initial target sample in some dimensions, including the migration background of participants [14]. Given that the age-20 wave of data collection includes participants who have had an ongoing relationship with z-proso and research participation experiences since the age of 7 years, the sample may also not be reflective of typical EMA participation pools. Our findings may also not generalize to participation willingness in other research designs, research questions, and data collection modes because participants may vary in their level of interest and concerns about taking part depending on what is asked of them and the topic under study. Some of the predictors or features were also measured with single items (eg, general health) and multi-item measures could have provided greater reliability or capture more nuances in the concepts. This could potentially improve prediction accuracy. We also did not measure the full range of concepts that may impact participation willingness (eg, the presence of medical conditions or participants’ own or family members’ involvement with research or medicine as a profession). In future studies, it will be important to replicate our findings in EMA studies with different methodological designs and target populations (eg, in clinical populations) to evaluate the extent to which findings generalize to different EMA settings. Finally, our approach was exploratory and focused on prediction, therefore, we used methods suited to this goal. Future studies focused on understanding and explaining EMA participation would provide important complementary information. These studies could focus on developing more parsimonious and interpretable models by, for example, using variable selection (eg, facilitated by lasso) to promote more spare solutions.

### Conclusions

Migration status emerged as the most important predictor of EMA study participation willingness, with social exclusion, prosociality, and tobacco use also significant predictors that could be targeted in strategies to reduce or adjust for selective participation. However, overall participation willingness was only weakly predicted, even when considering a broad span of respondent characteristics.

## Appendix

Multimedia Appendix 1 Supplementary materials on measures, overall descriptive statistics, descriptive statistics in complete-case sub-sample, logistic regression results for complete case analysis, and model evaluation metrics.

## Abbreviations

ADHD: attention deficit hyperactivity disorder
AUC: area under the curve
CART: classification and regression tree
D2M: Decades-to-Minutes
EMA: ecological momentary assessment
ISEI: International Socioeconomic Index of occupational status
RMSE: root mean squared error
SBQ: Social Behavior Questionnaire
Z-proso: Zurich Project on the Social Development from Childhood to Adulthood
