# Supplementary material for: Predicting Participation Willingness in Ecological Momentary Assessment of General Population Health and Behavior: Machine Learning Study
Source: J Med Internet Res. 2023 Aug 2;25:e41412. doi: 10.2196/41412 (PMC10433031; doi:10.2196/41412)
Supplement: Multimedia Appendix 1 [file jmir_v25i1e41412_app1.docx]

**Supplementary Materials**

**Measures**

Sociodemographic predictors were: *gender*, socioeconomic status based on the ISEI measure of *household occupational prestige* (Ganzeboom et al., 1992), *educational level* (1= incomplete compulsory school, 2=compulsory school, elementary vocational training, 3=domestic science course, 1-year of commerce school, 4= apprenticeship, 5=full time vocational school, 6= A-levels, 7=vocational high education, 8=technical school or vocational college, 9=vocational high school, higher specialized school, 10=University)*,* and *migration background* (1= at least one parent born in Switzerland, 2 = both parents born abroad) self-reported by youth at ages 11-12.

Mental health predictors were age 20 *ADHD symptoms, depression, anxiety, self-injury*, *suicidal ideation* and *psychosis-like symptoms*. ADHD symptoms, depression, and anxiety were measured with the Social Behavior Questionnaire (SBQ; Murray et al., 2017; Tremblay et al., 1991). ADHD was measured using 9 items (example item: ‘*You were restless, and struggled to sit still.*’), depression using 9 items (example: *‘I was sad without knowing why’*), and anxiety using 4 items (example: ‘*I was worried’*) . Psychosis-like symptoms were measured using an abbreviated version of the Community Assessment of Psychic Experiences Scale (Mark & Toulopoulou, 2016). Psychotic-like experiences were measured with six items (example: ‘*I heard voices when I was alone’*). For these predictors composite scores were derived by summing the individual item scores within the relevant scale/sub-scale.

Psychological well-being predictors were *hope, self-control, self-efficacy, optimism, general trust, perceived stress,* and *future orientation. Hope* was measured with an abbreviated version of the *Adult Hope Scale* (Snyder et al., 1991), which consists of 4 agency items (e.g., “I energetically pursue my goals”), and 4 pathways items (e.g., “There are lots of ways around any problem”). *Self-control* was measured by 10 items (e.g., “I often act on the spur of the moment without stopping to think”) which was an adapted version of Grasmick’s Low Self-control scale (Grasmick et al., 1993; Longshore et al., 1996), measuring impulsivity, risk-seeking, preference for physical over cognitive activity, temper, and self-centredness. *Self-efficacy* was measured with 5 items which was adapted from the 10-item scale proposed by Schwarzer & Jerusalem (1999) (e.g., “If there are difficulties, I find ways or means to overcome them”). *Optimism* was measured with 4 items (i.e., “I’m happy”, ”life is beautiful”, “I’m full of energy”, and “I laugh often”). *Perceived stress* was measured using 4 items (e.g., “I was unable to control the important things in my life”), an abbreviated version of the *Perceived Stress Scale* (Cohen, 1988)*. Future orientation* was measured with 5 items, covering the vividness of future (e.g., “easy to imagine future”), connectiveness with future (“level of connection between past/present with future”), and feelings about future (“feel about future self”).

Social well-being predictors were *social exclusion, social support from a trusted adult,* and *bullying victimisation.* Social support was measured with 5 items, including (e.g., “adults to talk about problems”, “adults I admire”, “discuss my problems with adults”, “adults I can trust”, “adult social support”). Social exclusion was measured with 6 items (e.g., “not feeling as part of society”, “being segregated”, “no chance in this society”, “feeling that others depreciate me”, “feeling alienated”, “feeling worthless for society”). *Bullying victimisation* was measured using the 4-item Zurich Brief Bullying Scale (ZBBS), which captures bullying victimisation in various forms, including physical and verbal aggression (A. Murray et al., 2019).

Physical predictors were *general health* and body mass index (BMI). General health was measured by a single item asking youth to rate their general health. *BMI* was derived from self-reported height and weight at age 20.

Prosocial trait predictors were *general trust, prosociality,* and *moral shame*. *Prosociality* was measured by 10 SBQ items, capturing both prosocial emotions (e.g., empathy) and helpful behaviours. *General trust* was measured using a 3-item (e.g., “most people can be trusted”) measure adapted from the *World Values Survey* (Johnson & Mislin, 2012). Moral shame was measured by 3 items (e.g., “ashamed after lying”, “feel bad when doing wrong”, and “guilty when done wrong”).

Anti-social trait predictors were age 20 *delinquency, bullying perpetration, aggression, substance use, psychopathy,* and *violent ideations*. *Delinquency* was measured by 24 items covering skipping work, stealing at school, stealing at work, stealing at home, shoplifting goods worth less than 50CHF, shoplifting goods worth more than 50CHF, vehicle theft, driving wo licence, illegal up/download, car/house burglary, drug dealing, cheating 100CHF+, driving on drugs, exceed speed limit, fare dodging, graffitti/tags, vandalism, carrying a weapon, sexual assault, urge to sexual acts, threat/extortion, robbery, death threat, assault. *Bullying perpetration* was measured using the 4-item ZBBS perpetration scale (A. Murray et al., 2019). Items were analogous to those in the victimisation scale but presented from a perpetrator’s perspective. *Substance use* was measured by four items capturing the substance use over the previous 12 months on a 6-point scale from never to daily (never, once, 2 to 5 times, 6 to 12 times monthly, 13 to 52 times weekly, and 53 to 365 times daily). One item captured the use of tobacco, one captured alcohol use (beer, alcopops), one captured alcohol use (spirits) and one captured cannabis use. *Violent ideations* were measured using the *Violent Ideations Scale expanded* (A. L. Murray et al., 2018; Urruela et al., 2021) capturing fantasies of committing acts of physical, indirect, and sexual violence across 17 items. *Aggression* was measured using 19 items from the SBQ, covering physical, indirect, proactive, and reactive aggression.

**Tables**

**Table S1: Descriptive Statistics**

| **Categorical predictors** | | | | | |
| --- | --- | --- | --- | --- | --- |
| **Gender** | Male =584, Female =599 | | | | |
| **Migration background** | At least one parent born in Switzerland=605,  Both parents born abroad=554 | | | | |
| **Education level** | Incomplete compulsory school= 64,  Compulsory school/elementary vocational training=201,  Domestic science course/1-year school of commerce=16,  Apprenticeship=248,  Full time vocational school=43,  A-levels=163,  Vocational high education=45,  Technical school of vocational college=38,  Vocational high school/higher specialised school =37,  University=270 | | | | |
| **Continuous predictors** | | | | | |
|  | **n** | **Mean** | **SD** | **min** | **max** |
| **ISEI** | 1068 | 47.32 | 19.57 | 16.00 | 90.00 |
| **Depression** | 1179 | 20.31 | 7.35 | 9.00 | 45.00 |
| **Anxiety** | 1180 | 9.61 | 3.69 | 4.00 | 20.00 |
| **Psychosis-like symptoms** | 1177 | 8.79 | 3.12 | 6.00 | 30.00 |
| **ADHD symptoms** | 1170 | 25.28 | 6.33 | 9.00 | 45.00 |
| **Hope** | 1177 | 2.94 | 0.45 | 1.00 | 4.00 |
| **Self-control** | 1179 | 2.07 | 0.42 | 1.00 | 3.80 |
| **Self-efficacy** | 1179 | 2.87 | 0.47 | 1.00 | 4.00 |
| **Optimism** | 1177 | 12.85 | 2.29 | 4.00 | 16.00 |
| **Stress** | 1180 | 11.28 | 3.72 | 4.00 | 20.00 |
| **Future Orientation** | 1174 | 18.03 | 4.21 | 5.00 | 30.00 |
| **Social Exclusion** | 1176 | 9.12 | 3.54 | 6.00 | 24.00 |
| **Social Support** | 1178 | 12.94 | 2.66 | 4.00 | 16.00 |
| **Bullying Victimisation** | 1179 | 1.37 | 0.47 | 1.00 | 5.25 |
| **BMI** | 1176 | 23.34 | 4.13 | 14.88 | 55.49 |
| **General health** | 1177 | 55.14 | 22.46 | 0.00 | 100.00 |
| **General trust** | 1180 | 2.34 | 0.67 | 1.00 | 4.00 |
| **Shame** | 1179 | 2.91 | 0.65 | 1.00 | 4.00 |
| **Prosociality** | 1168 | 38.17 | 5.62 | 12.00 | 50.00 |
| **Bullying perpetration** | 1180 | 1.32 | 0.44 | 1.00 | 6.00 |
| **Aggression** | 1169 | 27.97 | 6.89 | 19.00 | 72.00 |
| **Delinquency** | 1174 | 2.47 | 2.19 | 0.00 | 13.00 |
| **Violent Ideations** | 1177 | 19.47 | 5.02 | 17.00 | 62.00 |
| **Tobacco use** | 1177 | 3.68 | 1.92 | 1.00 | 6.00 |
| **Alcohol use (beer/wine/alcopops)** | 1177 | 3.72 | 1.39 | 1.00 | 6.00 |
| **Alcohol use (spirits)** | 1177 | 3.34 | 1.25 | 1.00 | 6.00 |
| **Cannabis use** | 1178 | 2.55 | 1.67 | 1.00 | 6.00 |

**Table S2: Descriptive statistics for complete case sub-sample**

| **Categorical predictors** | | | | | |
| --- | --- | --- | --- | --- | --- |
| **Gender** | male= 472; female=507 | | | | |
| **Migration** | At least one parent born in Switzerland=529  Both parents born abroad=450 | | | | |
| **Education** | Incomplete compulsory school=51; Compulsory school/elementary vocational training=164; domestic science course/1-year school of commerce=14; apprenticeship=214; full time vocational school=39; A-levels=141; vocational high school, higher specialised school=41; technical school or vocational college=32; vocational school/higher specialised school=34; university=249 | | | | |
| **Continuous predictors** | | | | | |
|  | **N** | **Mean** | **SD** | **Min** | **Max** |
| **ISEI** | 979 | 47.855 | 19.571 | 16.000 | 90.000 |
| **Depression** | 979 | 20.143 | 7.225 | 9.000 | 44.000 |
| **Anxiety** | 979 | 9.558 | 3.638 | 4.000 | 20.000 |
| **Psychosis** | 979 | 8.681 | 2.930 | 6.000 | 24.000 |
| **ADHD** | 979 | 25.269 | 6.194 | 9.000 | 45.000 |
| **Hope** | 979 | 2.945 | 0.448 | 1.000 | 4.000 |
| **Self-efficacy** | 979 | 2.882 | 0.467 | 1.000 | 4.000 |
| **Optimism** | 979 | 12.883 | 2.274 | 4.000 | 16.000 |
| **Stress** | 979 | 11.265 | 3.713 | 4.000 | 20.000 |
| **Future Orientation** | 979 | 18.057 | 4.153 | 5.000 | 29.000 |
| **Social Support** | 979 | 13.029 | 2.625 | 4.000 | 16.000 |
| **Social Exclusion** | 979 | 9.048 | 3.504 | 6.000 | 24.000 |
| **Bullying Victimisation** | 979 | 1.377 | 0.466 | 1.000 | 4.250 |
| **General Health** | 979 | 55.188 | 22.047 | 0.000 | 100.000 |
| **BMI** | 979 | 23.248 | 3.953 | 14.881 | 54.321 |
| **Trust** | 979 | 2.371 | 0.653 | 1.000 | 4.000 |
| **Shame** | 979 | 2.919 | 0.654 | 1.000 | 4.000 |
| **Prosociality** | 979 | 38.271 | 5.413 | 17.000 | 50.000 |
| **Bullying perpetration** | 979 | 1.320 | 0.415 | 1.000 | 3.750 |
| **Aggression** | 979 | 27.665 | 6.350 | 19.000 | 72.000 |
| **Delinquency** | 979 | 2.505 | 2.198 | 0.000 | 13.000 |
| **Violent Ideations** | 979 | 19.322 | 4.667 | 17.000 | 62.000 |

**Table S3: Logistic regression results for complete case analysis**

|  | **Estimate** | **SE** | ***p*** | **OR** |
| --- | --- | --- | --- | --- |
| **Intercept** | 0.681 | 0.435 | .118 | 1.975 |
| **Gender** | 0.064 | 0.040 | .115 | 1.066 |
| **Migration background** | -0.137 | 0.039 | <.001 | 0.872 |
| **ISEI** | 0.104 | 0.081 | .195 | 1.110 |
| **Education: Compulsory school/elementary vocational training** | 0.091 | 0.152 | .551 | 1.095 |
| **Education: Domestic science course/1-year school of commerce** | -0.018 | 0.081 | .825 | 0.982 |
| **Education: Apprenticeship** | -0.008 | 0.108 | .939 | 0.992 |
| **Education: Full time vocational school** | 0.034 | 0.087 | .693 | 1.035 |
| **Education: A-levels** | 0.005 | 0.109 | .963 | 1.005 |
| **Education: Vocational high education** | 0.022 | 0.115 | .848 | 1.022 |
| **Education: Technical school or vocational college** | 0.071 | 0.116 | .544 | 1.073 |
| **Education: Vocational high school/higher specialised school** | 0.080 | 0.090 | .377 | 1.083 |
| **ISEI** | -0.002 | 0.001 | .099 | 0.998 |
| **Depression** | 0.001 | 0.005 | .846 | 1.001 |
| **Anxiety** | 0.002 | 0.008 | .773 | 1.002 |
| **Psychosis-like symptoms** | 0.006 | 0.007 | .433 | 1.006 |
| **ADHD symptoms** | 0.003 | 0.004 | .468 | 1.003 |
| **Hope** | -0.093 | 0.056 | .100 | 0.911 |
| **Self-efficacy** | -0.033 | 0.051 | .513 | 0.967 |
| **Optimism** | 0.000 | 0.009 | .973 | 1.000 |
| **Stress** | 0.002 | 0.007 | .751 | 1.002 |
| **Future Orientation** | 0.006 | 0.004 | .190 | 1.006 |
| **Social Support** | 0.001 | 0.007 | .922 | 1.001 |
| **Social Exclusion** | -0.014 | 0.007 | .042 | 0.986 |
| **Bullying Victimisation** | 0.008 | 0.047 | .870 | 1.008 |
| **Health** | 0.001 | 0.001 | .361 | 1.001 |
| **BMI** | -0.015 | 0.025 | .549 | 0.985 |
| **BMI^2^** | 0.000 | 0.000 | .702 | 1.000 |
| **General Trust** | -0.008 | 0.028 | .773 | 0.992 |
| **Shame** | -0.004 | 0.028 | .897 | 0.996 |
| **Prosociality** | 0.009 | 0.004 | .016 | 1.009 |
| **Bullying Perpetration** | 0.067 | 0.053 | .205 | 1.069 |
| **Aggression** | -0.002 | 0.003 | .558 | 0.998 |
| **Delinquency** | 0.008 | 0.009 | .402 | 1.008 |
| **Violent ideations** | -0.002 | 0.004 | .639 | 0.998 |
| **Tobacco use** | -0.028 | 0.010 | .006 | 0.973 |
| **Alcohol use (beer/wine/alcopops)** | 0.017 | 0.020 | .383 | 1.017 |
| **Alcohol use (spirits)** | -0.004 | 0.022 | .838 | 0.996 |
| **Cannabis use** | 0.018 | 0.012 | .134 | 1.018 |

*Note.* Reference category for gender is ‘male’, reference category for migration status is ‘at least one parent born in Switzerland’, reference category for education is ‘incomplete compulsory school’

**Table S4**. Model evaluation metrics for performance comparison with tuning and cross-validation.

| Model | Data set | CV folds | Tuning parameters^a^ | Accuracy | AUC^b^ | Kappa | *F_1_* | Sensitivity | Specificity | PPV^c^ | NPV^d^ | Precision | | | Recall | CV^e^ AUC |
| --- | --- | --- | --- | --- | --- | --- | --- | --- | --- | --- | --- | --- | --- | --- | --- | --- |
| Logistic | Complete cases | 10 | Lambda=1 | 0.579 | 0.594 | 0.159 | 0.602 | 0.633 | 0.526 | 0.574 | 0.586 | | 0.574 | 0.632 | | 0.557 |
| CART^f^ | Complete cases | 10 | Tree depth=16 | 0.579 | 0.601 | 0.159 | 0.559 | 0.531 | 0.629 | 0.591 | 0.570 | | 0.591 | 0.531 | | 0.542 |
| Random forest | Complete cases | 10 | Features/split=2 | 0.518 | 0.585 | 0.035 | 0.552 | 0.592 | 0.443 | 0.518 | 0.518 | | 0.518 | 0.592 | | 0.569 |
| Logistic | Single imputation | 10 | Lambda=1 | 0.536 | 0.572 | 0.071 | 0.573 | 0.613 | 0.457 | 0.537 | 0.535 | | 0.537 | 0.613 | | 0.556 |
| CART | Single imputation | 10 | Tree depth=16 | 0.557 | 0.557 | 0.115 | 0.559 | 0.555 | 0.560 | 0.564 | 0.551 | | 0.564 | 0.555 | | 0.533 |
| Random forest | Single imputation | 10 | Features/split=2 | 0.549 | 0.576 | 0.095 | 0.598 | 0.664 | 0.431 | 0.545 | 0.556 | | 0.545 | 0.664 | | 0.562 |
| Logistic | Complete cases | 5 | Lambda=1 | 0.579 | 0.594 | 0.159 | 0.602 | 0.633 | 0.526 | 0.574 | 0.586 | | 0.574 | 0.633 | | 0.556 |
| CART | Complete cases | 5 | Tree depth=30 | 0.579 | 0.601 | 0.159 | 0.559 | 0.531 | 0.629 | 0.591 | 0.570 | | 0.591 | 0.531 | | 0.535 |
| Random forest | Complete cases | 5 | Features/spilt=2 | 0.590 | 0.613 | 0.179 | 0.608 | 0.633 | 0.546 | 0.585 | 0.596 | | 0.585 | 0.633 | | 0.568 |
| Logistic | Single imputation | 5 | Lambda=10 | 0.515 | 0.569 | 0.020 | 0.657 | 0.916 | 0.103 | 0.512 | 0.545 | | 0.512 | 0.916 | | 0.551 |
| CART | Single imputation | 5 | Tree depth=16 | 0.536 | 0.566 | 0.072 | 0.551 | 0.563 | 0.509 | 0.540 | 0.531 | | 0.540 | 0.563 | | 0.522 |
| Random forest | Single imputation | 5 | Features/split=2 | 0.562 | 0.570 | 0.121 | 0.605 | 0.664 | 0.457 | 0.556 | 0.570 | | 0.556 | 0.664 | | 0.557 |

^a^Parameter selected based on the highest AUC across models compared.

^b^AUC: area under the curve.

^c^PPV: positive predictive value.

**References**

Cohen, S. (1988). *Perceived stress in a probability sample of the United States.*

Donath, C., Bergmann, M. C., Kliem, S., Hillemacher, T., & Baier, D. (2019). Epidemiology of suicidal ideation, suicide attempts, and direct self-injurious behavior in adolescents with a migration background: A representative study. *BMC Pediatrics*, *19*(1), 1–15.

Ganzeboom, H. B., De Graaf, P. M., & Treiman, D. J. (1992). A standard international socio-economic index of occupational status. *Social Science Research*, *21*(1), 1–56.

Grasmick, H. G., Tittle, C. R., Bursik Jr, R. J., & Arneklev, B. J. (1993). Testing the core empirical implications of Gottfredson and Hirschi’s general theory of crime. *Journal of Research in Crime and Delinquency*, *30*(1), 5–29.

Johnson, N. D., & Mislin, A. (2012). How much should we trust the World Values Survey trust question? *Economics Letters*, *116*(2), 210–212.

Longshore, D., Rand, S. T., & Stein, J. A. (1996). Self-control in a criminal sample: An examination of construct validity. *Criminology*, *34*(2), 209–228.

Mark, W., & Toulopoulou, T. (2016). Psychometric properties of “community assessment of psychic experiences”: Review and meta-analyses. *Schizophrenia Bulletin*, *42*(1), 34–44.

Murray, A., Eisner, M., Ribeaud, D., Kaiser, D., McKenzie, K., & Murray, G. (2019). Validation of a brief self-report measure of adolescent bullying perpetration and victimisation: The Zurich Brief Bullying Scales (ZBBS). *Assessment*.

Murray, A. L., Eisner, M., & Ribeaud, D. (2018). Development and validation of a brief measure of violent thoughts: The Violent Ideations Scale (VIS). *Assessment*, *25*(7), 942–955.

Murray, A. L., Obsuth, I., Eisner, M., & Ribeaud, D. (2017). Evaluating longitudinal invariance in dimensions of mental health across adolescence: An analysis of the Social Behavior Questionnaire. *Assessment*, 1073191117721741.

Perret, L. C., Orri, M., Boivin, M., Ouellet-Morin, I., Denault, A.-S., Côté, S. M., Tremblay, R. E., Renaud, J., Turecki, G., & Geoffroy, M.-C. (2020). Cybervictimization in adolescence and its association with subsequent suicidal ideation/attempt beyond face-to-face victimization: A longitudinal population-based study. *Journal of Child Psychology and Psychiatry*, *61*(8), 866–874.

Schwarzer, R., & Jerusalem, M. (1999). Skalen zur erfassung von Lehrer-und schülermerkmalen. *Dokumentation Der Psychometrischen Verfahren Im Rahmen Der Wissenschaftlichen Begleitung Des Modellversuchs Selbstwirksame Schulen. Berlin: Freie Universität Berlin*, *144*.

Snyder, C. R., Harris, C., Anderson, J. R., Holleran, S. A., Irving, L. M., Sigmon, S. T., Yoshinobu, L., Gibb, J., Langelle, C., & Harney, P. (1991). The will and the ways: Development and validation of an individual-differences measure of hope. *Journal of Personality and Social Psychology*, *60*(4), 570.

Tremblay, R. E., Loeber, R., Gagnon, C., Charlebois, P., Larivee, S., & LeBlanc, M. (1991). Disruptive boys with stable and unstable high fighting behavior patterns during junior elementary school. *Journal of Abnormal Child Psychology*, *19*(3), 285–300.

Urruela, C., Booth, T., Eisner, M., Ribeaud, D., & Murray, A. L. (2021). Validation of an extended violent ideations scale to measure both non-sexual and sexual violent ideations. *European Journal of Psychological Assessment*.
